# Preoperative Radiotherapy and Local Recurrence in Grade 3 Soft Tissue Sarcoma: A Causal Bayesian Network Analysis

**DOI:** 10.3390/cancers18203269

**Published:** 2026-10-10

**Authors:** Andrea Valle, Bruno Fuchs, Mirko Birbaumer, Philip Heesen

**Affiliations:** 1 Lucerne School of Business, Lucerne University of Applied Sciences and Arts, 6002 Lucerne, Switzerland; andrea.valle@hslu.ch; 2 Swiss Sarcoma Network, LUKS Sarcoma-IPU, University Teaching Hospital of Lucerne, 6000 Lucerne, Switzerland; fuchs@sarcoma.surgery; 3 Faculty of Health Sciences and Medicine, University of Lucerne, 6002 Lucerne, Switzerland; 4 Kantonsspital Winterthur Sarcoma Center, 8401 Winterthur, Switzerland; 5 Faculty of Medicine, University of Zurich, 8006 Zurich, Switzerland; philip.heesen@uzh.ch

**Keywords:** soft tissue sarcoma, preoperative radiotherapy, local recurrence, causal Bayesian network, propensity score weighting, precision oncology, real-world data

## Abstract

Background: Estimating the effect of preoperative radiotherapy in grade 3 (G3) soft tissue sarcoma (STS) is difficult because treatment reflects clinical risk. We estimated whether local recurrence risk among treated patients would differ under surgery alone. Methods: We retrospectively analyzed a multicentre registry of 151 surgically treated G3 patients without chemotherapy; 99 received preoperative radiotherapy and 52 underwent surgery alone. Propensity score weighting targeted the average treatment effect among treated patients (ATT). The primary weighted interventional Bayesian network (BN) adjusted for age, metastasis, tumor size, depth, body location, and whoops surgery. Logistic regression and BN conditioning and intervention were reference analyses. Differential follow-up was assessed using a weighted Aalen–Johansen analysis. Results: Local recurrence occurred in 31 patients (20.5%). Weighting reduced the maximum absolute standardized mean difference from 0.866 to 0.139. Median bootstrap ATT estimates were +3.3 percentage points for conventional logistic regression, +5.2 for weighted logistic regression, and +1.37 for the weighted interventional BN. At 1 year, the weighted Aalen–Johansen risk difference was +0.83 percentage points, with a wide 95% confidence interval spanning benefit and harm. Conclusions: The weighted interventional BN produced a near-null estimate, but modest benefit or harm remained plausible. Treatment effect estimates were sensitive to the assumed causal structure, limiting definitive conclusions about radiotherapy benefit. Given the modest sample, limited treatment overlap, differential follow-up, and residual confounding, causal interpretation should remain cautious. These findings do not support changing established indications for preoperative radiotherapy, but this framework provides a transparent approach to evaluating treatment strategies in rare cancers.

## Simple Summary

Grade 3 soft tissue sarcoma is a rare and aggressive cancer. Radiotherapy is often given before surgery to patients with larger, deeper, or anatomically complex tumors, which makes it difficult to determine its effect from routine clinical data. We analyzed 151 patients, of whom 99 received preoperative radiotherapy and 52 underwent surgery alone. A causal Bayesian network was used to represent the clinical treatment pathway and to account for differences between the treatment groups. After adjustment, the estimated difference in local recurrence risk was small and close to zero. However, the uncertainty around the estimate remained compatible with modest effects in either direction, so the findings do not establish that preoperative radiotherapy has no effect on recurrence. Treatment effect estimates were sensitive to the assumed causal structure, limiting definitive conclusions about radiotherapy benefit. These findings should not be interpreted as supporting omission of preoperative radiotherapy or a change in established treatment recommendations. The study shows how clinically defined causal models can help separate treatment selection patterns from estimated treatment effects in rare cancers, although the findings require confirmation in larger datasets.

## 1. Introduction

Soft tissue sarcomas (STS) are rare mesenchymal malignancies accounting for less than 1% of adult cancers [1,2]. European data have shown geographic variation in age standardized incidence, ranging from approximately 3.3 cases per 100,000 persons per year in Eastern Europe to 4.7 per 100,000 in Northern Europe. The RARECARE project also estimated that approximately 280,000 people in the EU27 were living with a previous diagnosis of sarcoma at the beginning of 2003, of whom 83% had STS [3]. In Switzerland, registry data reported an age standardized STS incidence of 4.43 per 100,000 persons in 2011–2015, corresponding to approximately 330 new cases annually [2,4]. In the Swiss cohort covering 1996–2015, the median age at diagnosis was 62 years and 51.5% of patients were male [4]. Despite their rarity, STS comprise a heterogeneous group of malignancies with substantial differences in anatomical site, histology, grade, treatment pathways, and prognosis [1,2,5,6]. Histologic grade reflects tumor biological aggressiveness and in the widely used French Federation of Cancer Centers Sarcoma Group (FNCLCC) system, tumor differentiation, mitotic activity, and tumor necrosis are scored and combined to assign grade 1, 2, or 3. Grade 3 (G3), corresponding to the highest combined score, identifies tumors with particularly aggressive histologic features and a greater risk of local recurrence and distant metastatic progression [7]. In patients with G3 STS, local recurrence remains an important outcome after definitive oncologic surgery because it compromises local control and is influenced by tumor, treatment, and surgical factors [8,9]. Radiotherapy is an established component of local treatment for appropriately selected patients with high risk resectable STS, supported by randomized evidence that its addition to limb-sparing surgery improves local control [10,11]. Evidence comparing preoperative with postoperative radiotherapy informs treatment sequencing and toxicity rather than the effect of radiotherapy versus its omission [12]. Preoperative radiotherapy is commonly considered for larger, deeper, or tumors constrained by body location in which local failure risk is considered high or adequate surgical margins may be difficult to achieve [13,14,15,16,17]. Accordingly, management of high risk STS is individualized within a multidisciplinary setting integrating surgery, radiotherapy, and, in selected patients, systemic treatment [17].

Estimating its effect from observational data is difficult because treatment is assigned according to clinical risk rather than at random [18]. Characteristics that increase the probability of receiving preoperative radiotherapy may also increase local recurrence risk, creating confounding by diagnostic factors [19]. Consequently, unadjusted differences between preoperative radiotherapy and surgery alone may reflect disease severity at the treatment decision and be more related to treatment selection than to the effect of radiotherapy. Current causal inference practice formalises this through specification of the treatment strategies being compared, the target population to which inference applies, the time zero that aligns patients at a common starting point, the outcome definition, the follow-up period over which effects are assessed, and the target estimand [20,21,22]. This explicit specification can lead to different estimations even when analysing the same dataset, and it provides the foundation for selecting appropriate adjustment strategies and modelling approaches.

Registry data also preserve the temporal sequence of clinical care. Covariates precede treatment selection, while surgical findings such as margin status are observed only after treatment and definitive resection. Treating such downstream variables as covariates may block part of the treatment pathway or introduce post-treatment adjustment bias [23,24,25,26,27].

Bayesian networks (BNs) were selected because they provide a probabilistic graphical framework in which clinically motivated relationships among multiple variables can be represented explicitly while retaining a coherent joint probability model. Unlike an analysis centered on a single treatment coefficient, a BN can represent dependencies across baseline tumor characteristics, treatment selection, downstream surgical factors, and recurrence within the same model. This was particularly relevant to the present study because preoperative radiotherapy is embedded within a clinical pathway rather than acting as an isolated exposure. The graphical structure also allows prior clinical knowledge to be incorporated through predefined edge directions and structural constraints, while conditional probability distributions (CPDs) are estimated from the observed data. More generally, BNs can also accommodate informative probabilistic priors when suitable external evidence is available [28,29,30]. When the directed structure is given a causal interpretation, observational and interventional queries can be distinguished explicitly, allowing estimation of outcome probabilities after treatment is externally set to a specified value [23,31,32]. This was relevant because the clinical question concerned a structured treatment pathway rather than an isolated treatment coefficient. BNs have been applied in oncology for risk prediction, confounder identification, and treatment effect estimation [33]. In STS, previous BN and individualized prediction models have mainly focused on prognosis, dependency mapping, and decision support rather than explicit causal estimation of preoperative radiotherapy effects [34,35,36,37].

In this study, the clinical team specified and expert reviewed a causal BN representing the pathway from covariates through preoperative radiotherapy and pathological margin status to local recurrence. Propensity score weighting was used to reduce measured covariate imbalance and target the average treatment effect among treated patients [18,38,39]. The primary objective was to estimate how local recurrence risk among surgical G3 STS patients managed without chemotherapy and receiving preoperative radiotherapy would have differed had they instead undergone surgery alone. Alternative cohorts were also used to assess the robustness of the G3 estimate. By combining clinical pathway specification with causal inference, this analysis was intended to provide a transparent framework for evaluating precision treatment strategies in oncology, not a validated patient level decision support tool.

## 2. Materials and Methods

### 2.1. Study Design and Analytic Cohort

This retrospective, multicentre, causal modeling study comprised one primary causal analysis and supportive exploratory analyses. The primary cohort included patients with G3 STS undergoing definitive oncologic resection without chemotherapy. Chemotherapy recipients were excluded to define a specific and clinically interpretable comparison of preoperative radiotherapy versus surgery alone in the absence of systemic treatment. Chemotherapy is selectively administered according to additional clinical and prognostic factors and may differ in timing and regimen. Including these patients would therefore introduce an additional treatment pathway, potential confounding by prognosis, and combined treatment groups that could not be adequately characterized with the available data. The exclusion also reduced treatment heterogeneity and allowed the analysis to focus specifically on the local treatment pathway, although it limits the applicability of the findings to patients managed without chemotherapy.

The causal framework distinguished the time of treatment assignment from the origin of postoperative outcome follow-up. Time zero for treatment assignment was defined immediately before the clinical decision regarding preoperative radiotherapy, when all covariates used for confounding adjustment were available and before radiotherapy, definitive surgery, and pathological margin assessment. The compared treatment pathways were preoperative radiotherapy followed by definitive oncologic resection and definitive oncologic resection without preoperative radiotherapy. Patients who received radiotherapy only after definitive oncologic resection were excluded from the primary causal comparison because postoperative radiotherapy represents a distinct treatment pathway. Because preoperative radiotherapy necessarily introduced an additional interval before surgery, calendar time from the treatment decision to definitive resection was not assumed to be equivalent between the two pathways.

Preoperative radiotherapy was defined as documented receipt of radiotherapy before definitive oncologic resection, irrespective of dose or fractionation regimen. Dose, fractionation, and the interval from radiotherapy completion to surgery were summarized descriptively when these details were available but were not used to define treatment assignment. Completion of the planned radiotherapy course was considered supported when an end date or complete administered dose and fractionation were documented. Treatment assignment was based on documented receipt and did not require independently verifiable completion. The radiotherapy classification rules applied to the primary causal comparison are summarized in Table 1.

Broader clinically relevant cohorts were additionally analyzed to assess data support, treatment overlap, and model consistency. These analyses were exploratory and were not interpreted as confirmatory subgroup treatment effects.

The index surgery was the definitive oncologic resection. Whoops surgery was defined as an unplanned excision performed before the tumor was recognized as a sarcoma and without planned oncologic management. In the G3 cohort, 39 of 151 patients (25.8%) had undergone whoops surgery. Among the 36 patients for whom the dates of both the whoops procedure and definitive surgery were available, the whoops procedure occurred a median of 120 days (IQR, 64–161 days), or approximately 17 weeks, before definitive surgery. The median interval was 136 days among patients who subsequently received preoperative radiotherapy and 22 days among those who underwent surgery alone, reflecting the additional treatment interval associated with the preoperative radiotherapy pathway. Because whoops surgery occurred before subsequent treatment selection, it was treated as a baseline covariate in the causal model. Radiotherapy administered after the unplanned excision but before definitive repeat resection was classified as preoperative radiotherapy relative to definitive surgery.

**Figure 1:**
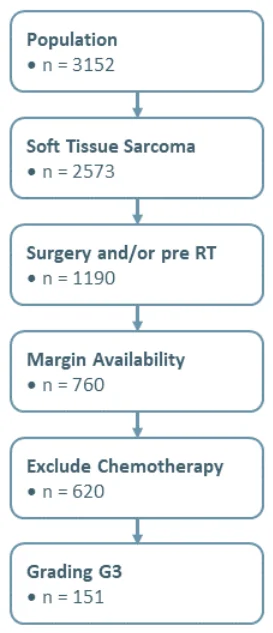
Sequential cohort selection flow diagram. Counts indicate the number of patients remaining after each selection step. The margin step denotes availability of a pathological margin assessment for the definitive resection; all recorded margins among otherwise eligible patients were R0 or R1, and no recorded R2 was excluded. R0 denotes a microscopically negative resection margin, and R1 denotes microscopic residual disease after macroscopically complete resection. The final G3 analytic cohort included 151 patients.

The primary G3 analytic cohort included 151 patients: 99 received preoperative radiotherapy and 52 underwent surgery alone. The sequential cohort selection process is shown in Figure 1. Starting from the full registry population, the cohort was restricted to patients with STS, then to those following one of the two treatment pathways under comparison: definitive oncologic resection with or without preoperative radiotherapy. Patients were required to have an available pathological margin assessment for the definitive resection. Among patients satisfying these pathway and data availability criteria, all recorded margins were R0 or R1, indicating macroscopically complete resection with either microscopically negative margins (R0) or microscopically positive margins (R1), respectively [40], no eligible patient had a recorded R2 of macroscopic positive margins. Patients receiving chemotherapy were excluded to retain the prespecified local treatment comparison, and the final restriction to histologic grade 3 defined the primary analytic cohort. The retained variables and their distributions are summarized in Table 2.

Age and initial tumor size were discretized into four and five quantile categories, respectively, to maintain observations across categories and limit sparse cells in the discrete conditional probability tables.

Local recurrence was selected as the primary endpoint because it directly reflects local control after surgery and preoperative radiotherapy and is less directly influenced than distant metastasis or survival by systemic disease biology and subsequent systemic treatments. Local recurrence was identified based on anatomical imaging findings. When imaging was inconclusive, recurrence was generally confirmed histologically by biopsy, while biopsy was not required when recurrence was unequivocal on imaging. The registry contained a single recorded date of local recurrence rather than separate imaging and biopsy dates. Therefore, when imaging and biopsy were performed at different time points, the analysis used the recurrence date recorded by the treating center without imposing a retrospective hierarchy between diagnostic modalities. Local recurrence was defined as a postoperative outcome, and its follow-up time therefore began at definitive oncologic resection rather than at the treatment decision time zero. In the binary BN analysis, the outcome indicated whether local recurrence was documented during the available postoperative follow-up, while the complementary time-to-event analysis used time since definitive resection.

The research question was whether a clinically constrained causal BN could represent the pathway from covariates through preoperative radiotherapy selection and pathological margin status to local recurrence in surgical G3 STS. The propensity weighted interventional BN using propensity weights truncated at the 99th percentile was the primary analysis. Conventional logistic regression, propensity weighted logistic regression, unweighted BN conditioning, unweighted BN intervention, and propensity weighted BN conditioning were reference analyses.

The analysis workflow was specified in advance as: (i) define the treatment decision time zero, postoperative outcome time origin, and estimand, (ii) classify treatment relative to definitive oncologic surgery, (iii) restrict adjustment to variables available before treatment assignment, (iv) assess overlap and covariate balance, (v) fit the weighted BN and reference models, (vi) report the model specific distributions, and (vii) interpret all contrasts in relation to support, graph assumptions, and model dependence.

### 2.2. Treatment Effect Estimation

Let *X* denote covariates measured by the treatment decision time zero, *T* denote the treatment pathway, and ${\mathrm{LR}}_{\mathrm{post}}$ denote local recurrence after definitive oncologic resection. Here, T=1 represented preoperative radiotherapy followed by definitive resection and T=0 represented definitive resection without preoperative radiotherapy. The treatment effect of interest was the average treatment effect among treated patients (ATT) in the preoperative radiotherapy pathway. The ATT was selected because the clinical question concerned surgically treated patients who received preoperative radiotherapy: specifically, how their postoperative local recurrence risk would have differed under the pathway of definitive surgery without preoperative radiotherapy.

$$\mathrm{ATT}=E_{X|T=1}\left[P\left({\mathrm{LR}}_{\mathrm{post}}=1|\mathrm{do}\left(T=1\right),X\right)-P\left({\mathrm{LR}}_{\mathrm{post}}=1|\mathrm{do}\left(T=0\right),X\right)\right],$$

Causal time zero was defined immediately before the clinical decision regarding preoperative radiotherapy, when the baseline covariates were measured. At this time, do(T=1) represents assignment to preoperative radiotherapy followed by definitive surgery, while do(T=0) represents assignment to definitive surgery without preoperative radiotherapy. The intervention therefore represents setting one of the two treatment pathways rather than merely conditioning on observed treatment. The expectation over X∣T=1 standardizes the contrast to the baseline covariate distribution of patients who received preoperative radiotherapy. The analysis was restricted to patients who subsequently underwent definitive surgery. Because local recurrence was defined as a postoperative outcome, its event time scale began at surgery; however, the surgery date did not replace the treatment decision time zero. The interval between treatment assignment and surgery remained part of the treatment pathway but was not counted as postoperative recurrence follow-up. Consequently, the estimand concerns postoperative local recurrence among surgically treated patients and does not estimate treatment effects on reaching surgery or on events occurring before surgery. The ATT was defined as recurrence risk under preoperative radiotherapy minus recurrence risk under surgery alone; negative ATT values indicate lower estimated recurrence risk with preoperative radiotherapy, while positive values indicate higher estimated risk.

### 2.3. Clinically Specified Causal BN

The clinical team specified and expert reviewed the BN structure a priori using domain knowledge and temporal ordering [41]. Nodes represented registry variables, and directed edges represented the assumed sequence from diagnosis through treatment selection and surgical findings to local recurrence. Thus, prior clinical knowledge entered the analysis primarily through the predefined network structure, structural constraints, and edge directions rather than through informative numerical priors; the conditional probability distributions were subsequently estimated from the observed data. The graph was not interpreted as causal solely because it was directed, since causal interpretation depends on the clinical specification of the variables, edge directions, and treatment timeline.

#### Causal Hypothesis and Assumptions

The graph was interpreted as an explicit clinical hypothesis about the process generating the observed data. An edge A→B indicated that *A* was assumed to precede and potentially directly influence *B* within the specified pathway. For example, tumor size could influence both radiotherapy selection and recurrence, while margin status occurred after treatment and surgery and could not influence the preceding radiotherapy decision.

The directed acyclic graph was a predefined causal model rather than proof that each edge represented a causal relationship. Its structure was specified a priori from clinical knowledge and temporal ordering rather than learned from the observed data. Statistical dependence among the baseline covariates was evaluated to identify strong associations and potentially redundant information. This diagnostic assessment did not require the covariates to be mutually or marginally independent, because baseline characteristics may be associated while representing distinct aspects of disease presentation and treatment selection.

The graph defined the assumed temporal and clinical relationships among the variables. The absence of an edge represented the absence of a direct dependency within the specified model, conditional on the other variables included in the graph [23]. Because the graph was clinically predefined rather than discovered using conditional independence tests, the assessment of dependence among covariates was not used to learn or modify the graph structure.

The assessment of dependence among measured covariates is distinct from conditional exchangeability, which concerns treatment assignment and potential outcomes. For the radiotherapy contrast, conditional exchangeability was expressed as

$$T⊥\{{\mathrm{LR}}_{\mathrm{post}}\left(1\right),{\mathrm{LR}}_{\mathrm{post}}\left(0\right)\}|X,$$

where *T* denotes the preoperative radiotherapy plus surgery or surgery without preoperative radiotherapy pathway, ${\mathrm{LR}}_{\mathrm{post}}\left(t\right)$ is the potential postoperative local recurrence outcome under pathway *t*, and *X* is the set of measured baseline covariates used for confounding control. Interventional interpretation additionally assumed consistency, positivity, and adequate capture in *X* of the main common causes of pathway selection and postoperative local recurrence [23,42]. These assumptions cannot be fully verified. Residual confounding from unmeasured or imperfectly measured treatment determinants, selection associated with restriction to patients who underwent definitive surgery, and misspecification of the graph structure therefore cannot be excluded.

The temporal classification of each variable and its inclusion in the propensity score and BN models are summarized in Table 3.

Pathological margin status was included in the BN as a downstream surgical finding but excluded from the propensity score model because it was observed after treatment and definitive surgery. Treating it as a covariate could block part of the treatment pathway or introduce post-treatment adjustment bias.

After fixing the qualitative graph, the BN factorizes the joint distribution as:

$$P\left(V_{1},V_{2},\ldots ,V_{k}\right)=\prod_{j=1}^{k}P\left(V_{j}|\mathrm{Pa}\left(V_{j}\right)\right),$$

where $\mathrm{Pa}(V_{j})$ denotes the parent variables of node $V_{j}$. Each conditional probability table can therefore be interpreted as the probability of a clinical variable given its preceding variables in the graph.

**Figure 2:**
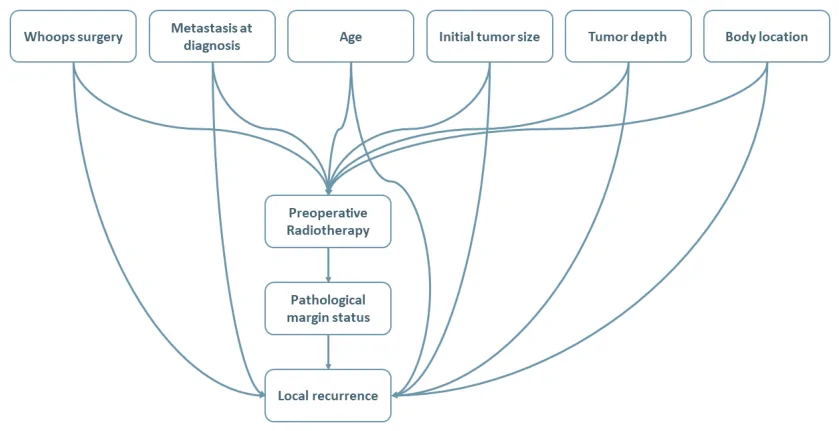
BN structure used for the analysis. Each baseline covariate has directed edges to preoperative radiotherapy and local recurrence. Preoperative radiotherapy points to pathological margin status, which in turn points to local recurrence. No direct edge from preoperative radiotherapy to local recurrence was specified.

Figure 2 shows the BN used to represent the main clinical pathway. The graph summarizes the covariates, treatment assignment, margin status, and local recurrence, while preserving the temporal ordering required for the analysis. No direct edge from preoperative radiotherapy to local recurrence was specified in the primary BN. Accordingly, within the prespecified graph, any effect of radiotherapy on local recurrence was assumed to operate through the downstream pathological margin status pathway. This does not mean that margin status necessarily reflects all biological mechanisms through which radiotherapy can influence local control, but it reflects the pathway represented by the variables available in the registries. Although a direct radiotherapy to local recurrence edge is clinically plausible, adding treatment as another parent of local recurrence approximately doubled the local recurrence CPD parameterization and created still sparser parent configurations. It was therefore evaluated as a graph structure sensitivity analysis.

### 2.4. Propensity Score Weighting

To address measured confounding by indication, propensity score weighting was used to reweight surgery alone patients toward the measured baseline covariate distribution of patients receiving preoperative radiotherapy. Propensity scores were estimated by logistic regression using age, metastasis at diagnosis, initial tumor size, tumor depth, body location, and whoops surgery status. Only variables available before treatment assignment were eligible for inclusion; post-treatment variables, including pathological margin status, were excluded to avoid adjustment for variables potentially lying on the treatment pathway. Missing covariate values were retained as an explicit category.

Histologic subtype was recorded and reported descriptively because of its potential influence on treatment selection and local recurrence. Its inclusion in the propensity score model was examined in a sensitivity specification but substantially reduced empirical treatment overlap and decreased the effective sample size from 80.56 to 52.98. Sparse histology combinations, particularly in the surgery alone group, indicated limited empirical support for estimating the surgery alone counterfactual among all patients receiving preoperative radiotherapy after histological adjustment. Histology was therefore not included in the primary propensity score or causal adjustment model. This decision was based on limited positivity and data support rather than an assumption that histology was clinically unimportant, and residual confounding by histological subtype cannot be excluded.

The propensity score was defined as

e(X)=P(T=1∣X).

Thus, patients were weighted as follows:

$$w_{i}=\left\{\begin{array}{cc} 1, & T_{i}=1,\\ \frac{e(X_{i})}{1-e(X_{i})}, & T_{i}=0. \end{array}\right.$$

Patients receiving preoperative radiotherapy therefore defined the target population, while surgery alone patients were reweighted to resemble their measured covariate distribution [43]. Under consistency, positivity, conditional exchangeability given *X*, and the specified graph, the resulting contrast estimates recurrence risk among patients selected for preoperative radiotherapy under radiotherapy versus surgery alone.

Covariate balance was assessed using standardized mean differences (SMDs) to quantify residual imbalance in the analytic sample, not to test equality of population parameters. Effective sample size, propensity score overlap, and the weight distribution were additionally examined to assess information retained after weighting, empirical positivity, and the influence of extreme weights [42,44].

### 2.5. Time-to-Event Analysis

Because postoperative follow-up differed between treatment groups, a complementary one-year time-to-event analysis was performed in the same ATT target population. Causal time zero remained the treatment decision, while postoperative event time was measured in years from definitive surgery. Local recurrence was the event of interest, death without prior recurrence was a competing event, and loss to follow-up before 1 year was censoring.

A treatment-stratified ridge-penalized Cox model estimated $G(t^{-}|T,X)$, the probability of remaining uncensored immediately before time *t*, using the baseline covariates from the propensity model. For 0≤t≤1 year, the censoring and combined analysis weights were

$$w_{i}^{\mathrm{IPCW}}\left(t\right)=\frac{1}{\hat{G}\left(t^{-}|T_{i},X_{i}\right)},\;w_{i}\left(t\right)=w_{i}\;w_{i}^{\mathrm{IPCW}}\left(t\right),$$

where $w_{i}$ is the ATT weight defined above. The combined weights were applied to the Aalen–Johansen estimator. Interpretation required conditional independent censoring given the modeled covariates, censoring positivity, correct model specification, consistency, and no unmeasured treatment–outcome confounding.

The primary time-to-event estimand was the one-year ATT risk difference

$$\Delta \left(1\;\mathrm{year}\right)={\hat{F}}_{\mathrm{LR},w}^{\mathrm{RT}}\left(1\;\mathrm{year}\right)-{\hat{F}}_{\mathrm{LR},w}^{\mathrm{SA}}\left(1\;\mathrm{year}\right).$$

Negative values favor preoperative radiotherapy. A treatment stratified patient level bootstrap with 1000 repetitions provided 95% percentile confidence intervals. The censoring model was refitted in each repetition; the propensity model and ATT weights were held fixed.

### 2.6. Interventional BN and Model Comparisons

BN parameters were learned by estimating the conditional probability tables with the expectation-maximization (EM) algorithm [45]. Propensity score weights were incorporated during EM so that the fitted conditional probability tables reflected the weighted population. In this weighted population, preoperative radiotherapy patients retained weight 1, while surgery alone patients were reweighted according to their propensity score to resemble the covariate distribution of the preoperative radiotherapy group.

BN analyses were implemented using pgmpy version 1.0.0.

The EM algorithm provided a consistent numerical method for estimating the weighted conditional probability tables using weighted sufficient statistics. Convergence was assessed using the weighted log-likelihood, and iterations continued until the relative change fell below the predefined threshold.

The fitted network quantified the predefined graph using clinically interpretable variable categories. For interventional queries, preoperative radiotherapy was treated as the intervention node. Under the intervention operator do(T=t), incoming edges into the treatment node were removed, treatment was fixed at T=1 or T=0, and the downstream probability of local recurrence was recomputed over the post-intervention graph [23,31].

The propensity weighted interventional BN was the primary model. Conventional logistic regression, weighted logistic regression, unweighted BN conditioning, unweighted BN intervention, and weighted BN conditioning were reference analyses. Logistic regression was included as a conventional reference approach for the binary local recurrence outcome. All contrasts represented recurrence risk under preoperative radiotherapy minus recurrence risk under surgery alone. BN conditioning used P(LR∣T=t,X) and was interpreted as an observational risk difference, while intervention used P(LR∣do(T=t),X) and targeted the ATT among patients receiving preoperative radiotherapy. Conditioning describes outcome probabilities among patients observed with a given treatment, while intervention represents the counterfactual treatment assignment required by the causal estimand.

Sampling uncertainty in the primary cohort comparison was quantified using a nonparametric bootstrap. In each replicate, 151 patients were sampled with replacement from the original cohort. Each logistic regression model was refitted to the bootstrap sample. For the BN analyses, the propensity score model was reestimated, weights were reconstructed, all conditional probability tables were refitted using the corresponding bootstrap sample, and the weights were applied only to the weighted BN specifications. The clinically prespecified DAG structure was held fixed. The 2.5th and 97.5th percentiles of each bootstrap treatment effect distribution were reported as a 95% percentile bootstrap confidence interval (CI).

For the discretization, clinical scenario, edge marginalization, and alternative fitting cohort analyses, repeated CPD perturbation was retained as a parameter sensitivity analysis rather than as an estimator of sampling uncertainty. Each conditional probability vector in every fitted CPD was perturbed independently by drawing from a Dirichlet distribution. For a fitted probability vector $\hat{p}$, the Dirichlet parameters were defined as $\alpha_{k}=\max\left(10{\hat{p}}_{k},1\right)$. The nominal concentration of 10 controlled the magnitude of the perturbation, while the minimum parameter of 1 prevented degenerate draws for rare or fitted zero-probability states and deliberately allowed such states to receive nonzero probability. For each of B=2000 perturbed sets of CPDs, the interventional risks and corresponding treatment effect estimate were recomputed while the graph structure, propensity score estimates, and weights were held fixed. The 2.5th and 97.5th percentiles of the resulting estimates were reported as central 95% CPD perturbation intervals. These intervals quantify sensitivity to the specified BN parameter perturbations; they are not frequentist confidence intervals or Bayesian posterior credible intervals and do not capture uncertainty from patient sampling, propensity score estimation, weighting, or graph specification.

### 2.7. Sensitivity and Robustness Analyses

As a sensitivity analysis for discretization, age and initial tumor size were recategorized using clinically interpretable thresholds. Age was categorized as <65, 65–74, and ≥75 years, and tumor size as ≤5, >5–10, >10–15, and >15 cm. The BN analysis was then repeated using the CPD perturbation procedure described above.

Two sensitivity analyses assessed large propensity weights and limited treatment overlap. The primary weighted interventional BN analysis used weights truncated at the 99th percentile, it was repeated using untruncated weights and, separately, after restricting the cohort to the empirical region of common propensity score support. Sampling uncertainty was quantified using the same nonparametric bootstrap as in the primary analysis. The common support analysis targets a narrower population of treated patients with empirically supported comparisons.

A graph structure sensitivity analysis added a direct edge from preoperative radiotherapy to local recurrence ($T\to {\mathrm{LR}}_{\mathrm{post}}$) while retaining all other nodes and edges from the prespecified BN. This edge is clinically plausible because radiotherapy may influence local control through biological mechanisms not captured by pathological margin status. Adding radiotherapy as another parent of local recurrence approximately doubled the local recurrence CPD parameterization by splitting every existing parent configuration according to treatment status. Given the modest cohort and number of recurrence events, this produced sparser conditional configurations and was expected to reduce parameter stability. The alternative graph was evaluated using the same nonparametric bootstrap procedure as the primary model, including reestimation of the propensity score, ATT weights, and all CPDs within each replicate.

Exploratory scenario analyses were performed by conditioning the fitted primary G3 models on clinically relevant characteristics, including whoops surgery status, absence of metastasis at diagnosis, tumor depth, body location, and tumor size. These analyses assessed sensitivity of the estimation contrast to clinically relevant patient profiles and were not interpreted as confirmatory subgroup treatment effects.

Edge sensitivity was evaluated through marginalization. For each clinically specified edge X→Y, the parent *X* was marginalized out of the conditional probability table of *Y*:

$$P\left(Y|Z\right)=\sum_{x}P\left(Y|X=x,Z\right)P\left(X=x|Z\right),$$

where *Z* denotes the remaining parents of *Y*. The remaining graph structure and fitted parameters were unchanged. The resulting ATT change quantified sensitivity to the modeled dependency and was not interpreted as the causal effect of the individual edge.

Robustness analyses assessed whether the G3 treatment contrast depended on the model fitting population. Propensity score models and BN parameters were independently refitted in alternative cohorts defined by tumor depth, body location, tumor size, whoops surgery status, and metastasis at diagnosis. Each fitted model was then evaluated conditional on G3 grade, preserving the primary research question.

## 3. Results

### 3.1. Cohort Characteristics

The primary G3 cohort included 151 patients: 99 received preoperative radiotherapy and 52 underwent surgery alone. Treatment allocation varied across tumor size and depth strata, supporting the need for adjustment. Local recurrence was documented in 31 patients (20.5%): 10 of 52 treated with surgery alone (19.2%) and 21 of 99 receiving preoperative radiotherapy (21.2%). The distributions and completeness of the variables included in the primary analysis are presented in Table 2. These unadjusted recurrence proportions were not interpreted causally because radiotherapy was preferentially assigned to patients with higher perceived local failure risk.

Among patients receiving preoperative radiotherapy with evaluable treatment details, the most frequent regimens were 50 Gy in 25 fractions (42/81, 51.9%) and 25 Gy in 5 fractions (32/81, 39.5%). The median interval between radiotherapy completion and definitive surgery was 42 days (IQR, 19–55 days; n=82). Completion of the planned course was supported by a documented end date or complete administered dose and fractionation for 96 of 99 patients (97.0%); completion remained unverifiable for 3 of 99 patients because treatment details were insufficient.

Median follow-up from diagnosis was 0.57 years (IQR, 0.18–2.62) after surgery alone and 2.76 years (IQR, 1.21–4.58) after preoperative radiotherapy. Among patients with recurrence, median time from diagnosis to local recurrence was similar between groups: 1.79 and 1.78 years, respectively.

When follow-up was measured from definitive surgery, consistently with the postoperative time-to-event analysis, median postoperative follow-up was 2.51 years (IQR, 1.03–4.25) after preoperative radiotherapy and 0.53 years (IQR, 0.10–2.78) after surgery alone.

### 3.2. Propensity Score Diagnostics

**Figure 3:**
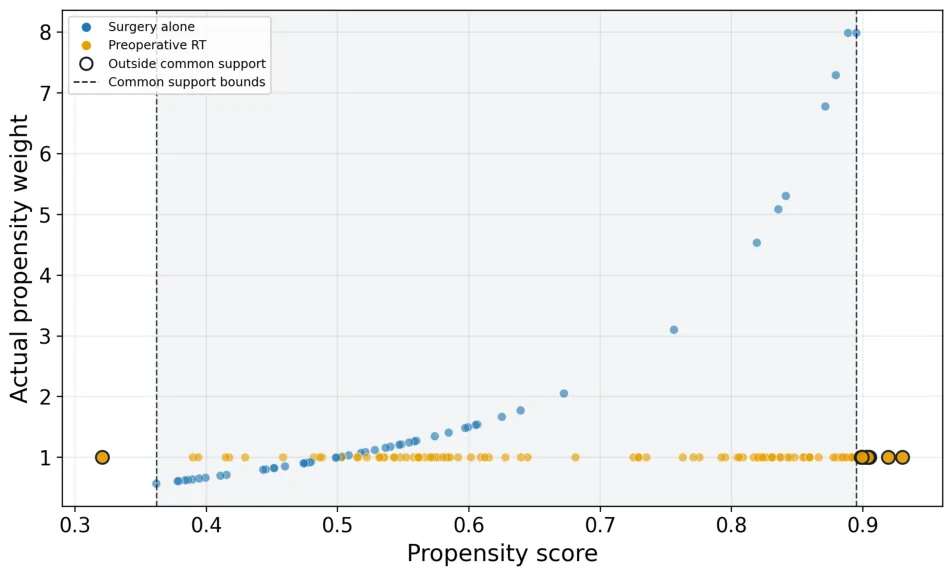
Relationship between the estimated propensity score *e* and the propensity weight. Preoperative radiotherapy patients have weight 1, while surgery alone patients are weighted by e(X)/(1−e(X)) to represent the covariate distribution of the preoperative radiotherapy group. Bounds are set in order to mark the patients outside the coverage.

Figure 3 and Table 4 summarize the weighting diagnostics. Surgery alone weights increased with the estimated probability of receiving radiotherapy, giving greater weight to patients who more closely resembled the treated population [44].

Weighting substantially improved measured balance. The maximum absolute standardized mean difference decreased from 0.866 to 0.139, the mean absolute standardized mean difference from 0.329 to 0.090, and the number of adjustment covariates with an absolute standardized mean difference above 0.15 from 6 of 8 to 0 of 8. The effective sample size (ESS) was 80.56. In the sensitivity propensity score specification that additionally included histologic subtype, sparse histology combinations reduced the ESS to 52.98, indicating substantially weaker empirical support for the adjusted ATT.

The empirical common support range was 0.362–0.895, with 10 patients outside common support. The median propensity weight was 1.000, the 99th percentile was 7.641, and the maximum was 7.989. Consequently, limited overlap was localized primarily at the extremes of the covariate distribution [42,46]. The weighted estimates were therefore interpreted within the observed support and conditional on the measured covariates and propensity score model.

Using untruncated weights and restricting the analysis to common support produced bootstrap median ATT estimates similar to the primary analysis (Table 5). All three bootstrap confidence intervals included zero. The common support restriction excluded 10 treated patients and therefore estimates the ATT in a narrower population of treated patients with better supported treatment comparisons.

### 3.3. Model Comparison

**Figure 4:**
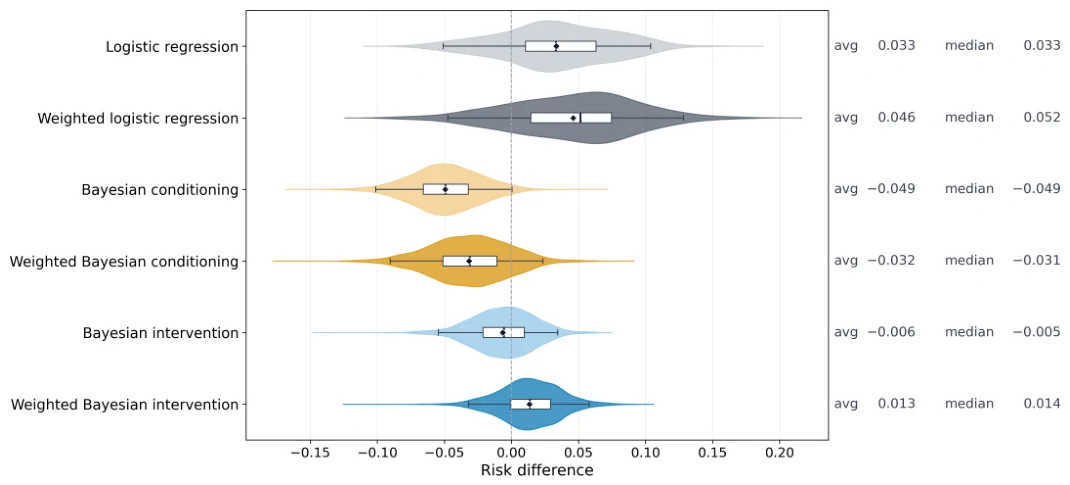
Bootstrap distributions for the six model specifications. In each BN bootstrap replicate, 151 patients were sampled with replacement, the propensity score model was reestimated, ATT weights were reconstructed and truncated at the 99th percentile for the primary weighted analysis, and the conditional probability tables were refitted, while the clinically prespecified DAG structure remained fixed. Propensity weights were applied only to the weighted model specifications. BN conditioning summarizes an observational risk difference, whereas BN intervention targets the ATT. The 2.5th and 97.5th percentiles define 95% percentile bootstrap confidence intervals. Diamonds indicate bootstrap means, boxes show medians and interquartile ranges, whiskers show the 95% percentile intervals; and violins show the bootstrap distributions. Positive values indicate higher estimated local recurrence risk under preoperative radiotherapy, while negative values indicate lower estimated risk.

Figure 4 compares the bootstrap distributions for all models. These distributions quantify sampling uncertainty while incorporating model refitting within each replicate; for the weighted models, the propensity score model and weights were also reestimated. Expressed in percentage points (pp), conventional logistic regression produced a bootstrap median ATT of +3.3 pp, while the weighted logistic regression median was +5.2 pp. For the BN analyses, the bootstrap median and 95% percentile interval were −4.92 pp (−10.11 to +0.05) for conditioning, −0.54 pp (−5.45 to +3.46) for intervention, −3.09 pp (−9.03 to +2.36) for weighted conditioning, and +1.37 pp (−3.19 to +5.81) for weighted intervention. The corresponding original sample estimates, reported for reference, were −5.88, −0.64, −3.78, and +2.37 pp, respectively.

The interventional BN estimates were therefore closer to the null than the regression estimates, while the conditional BN estimates were negative. This difference may reflect the explicit causal structure of the BN, in which intervention is propagated through the predefined clinical pathway, including downstream margin status, as well as the discretization of continuous variables. All four BN bootstrap intervals included zero, indicating that the data were compatible with modest reductions or increases in risk. The primary weighted interventional BN had a bootstrap median ATT of +1.37 pp and an original sample estimate of +2.37 pp, with its 95% percentile bootstrap CI of −3.19 to +5.81 pp should not be interpreted as evidence of equivalence or absence of a treatment effect.

Using the clinically defined categories, the weighted interventional BN yielded a median treatment contrast of +1.69 pp, with a central 95% CPD perturbation interval of −3.28 to +7.38 pp. Across the alternative clinical cut-point specifications, median estimates ranged from +1.37 to +2.49 pp, and the corresponding central 95% CPD perturbation interval for every specification included zero. Thus, the interpretation remained close to the null and was not materially altered by the choice of discretization thresholds.

Because logistic regression and BNs represent different modeling frameworks, their results were interpreted separately. Logistic regression modeled local recurrence as a function of treatment and measured covariates, while the BNs modeled the joint probability distribution according to the predefined causal and temporal clinical structure. The estimates should therefore be understood as results obtained under different modeling assumptions rather than as directly interchangeable implementations of the same model.

### 3.4. Direct Edge Graph Sensitivity

Adding the direct $T\to {\mathrm{LR}}_{\mathrm{post}}$ edge left the conditional estimates similar to those from the primary graph but materially changed the interventional estimates (Table 6). The bootstrap median was −5.16 pp for unweighted conditioning and −3.19 pp for weighted conditioning. In contrast, the interventional medians shifted to −14.33 pp without weighting and −12.26 pp with weighting, compared with −0.54 pp and +1.37 pp, respectively, in the primary graph without the direct edge. The interventional bootstrap intervals also became substantially wider, consistent with the sparse local recurrence parent configurations created by adding treatment as another parent.

Although three of the four percentile intervals excluded zero, these estimates should not be interpreted as robust evidence of a protective effect. The sensitivity specification approximately doubled the local recurrence CPD parameterization without adding observations, leaving fewer observations within each parent configuration. The pronounced shift and increased uncertainty in the interventional results therefore demonstrate dependence on graph specification and limited parameter stability in this dataset.

Table 7 shows the separate CPD perturbation scenario sensitivity analysis for clinically relevant subgroups in which preoperative radiotherapy may plausibly be considered for local control. Unlike the bootstrap in Figure 4, this analysis held the fitted propensity scores, ATT weights, and graph structure fixed and summarized parameter perturbations around each fitted BN. In the primary cohort, the weighted BN perturbation distribution median was +1.6 pp under conditioning (central 95% CPD perturbation interval, −6.4 to +7.7) and +1.6 pp under intervention (central 95% CPD perturbation interval, −5.3 to +9.3). Across scenarios, the location and spread of the perturbed CPD risk difference distributions varied according to the subgroup definition and BN specification, indicating that the estimated treatment contrast was sensitive to changes in the covariate distribution and model structure.

The weighted BN interventional distributions were consistently close to the null and generally closer to it than the corresponding conditional estimates. In the primary cohort, weighted conditioning and intervention were closely aligned, while larger differences appeared in several restricted scenarios. Conditional estimates therefore reproduced clinically recognizable treatment selection patterns, but these patterns were attenuated when treatment was set by intervention rather than observed within the clinical pathway.

Several clinically interpretable patterns were observed. Weighted interventional estimates remained close to the null among patients with and without previous whoops surgery, after excluding metastasis at diagnosis, and across tumor depth and body location scenarios. Conditional estimates were more negative in several of these groups, but these patterns shifted toward the null or became slightly positive under intervention and, for deep extremity tumors, occurred in cohorts with very limited surgery alone data support. In superficial sarcomas, both weighted contrasts remained close to the null and their central 95% CPD perturbation intervals crossed zero.

Tumor size scenarios further illustrated the difference between conditioning and intervention. Conditional estimates varied across size bins, whereas the weighted interventional estimates remained close to the null and were slightly positive. No consistent pattern of increasing radiotherapy benefit was therefore observed with increasing tumor size.

The scenario distributions were heterogeneous and generally crossed zero; all central 95% CPD perturbation intervals for the weighted interventional estimates included zero. The scenario analysis shows that simplified subgroup definitions can produce unstable and sometimes contradictory treatment contrast distributions, particularly in small cohorts with limited treatment overlap or recurrence events. This reinforces the importance of interpreting the primary multivariable weighted BN separately from the exploratory subgroup scenarios. The scenarios are useful for evaluating sensitivity to treatment selection, case mix, and model structure, but they do not provide sufficient evidence to identify subgroups that benefit from or are harmed by preoperative radiotherapy.

### 3.5. Time-to-Event Results

To address differential postoperative follow-up, local recurrence was evaluated with a weighted Aalen–Johansen estimator incorporating time varying inverse probability of censoring weights (IPCW). At 1 year, cumulative incidence was 5.89% under preoperative radiotherapy and 5.07% under surgery alone, a risk difference of +0.83 pp (95% CI, −9.88 to +8.51). The adjusted estimates were similar, and the confidence interval included zero and remained compatible with lower or higher recurrence under preoperative radiotherapy.

**Figure 5:**
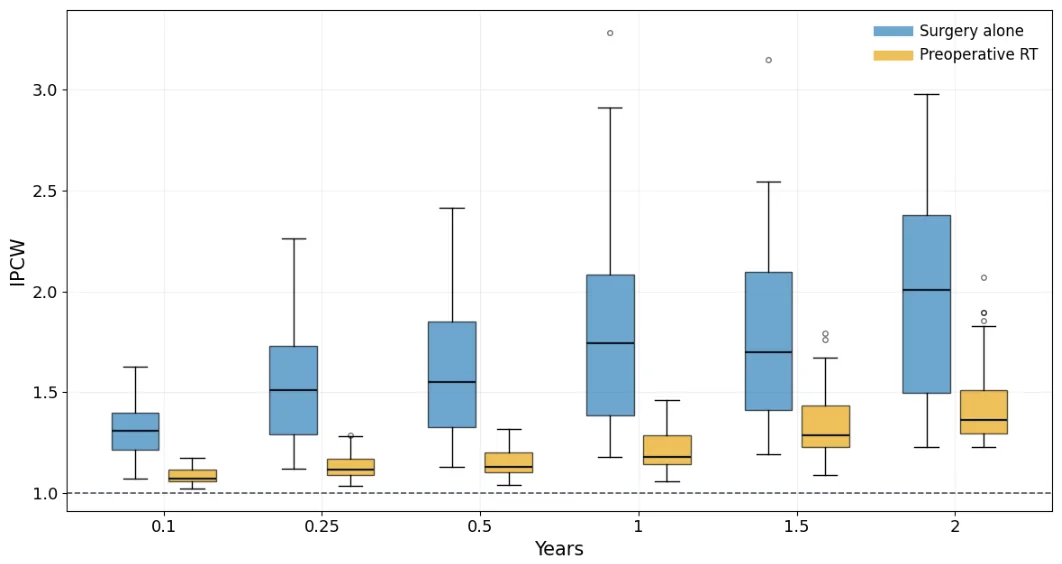
Distribution of IPCW by treatment group over follow-up. The dashed horizontal line marks a weight of 1.

At 1 year, 69 radiotherapy patients and 21 surgery alone patients remained at risk. IPCW values were moderate, but the combined weights reduced the pooled effective sample size to 28.129 (Figure 5; Table 8).

### 3.6. Edge Sensitivity

**Figure 6:**
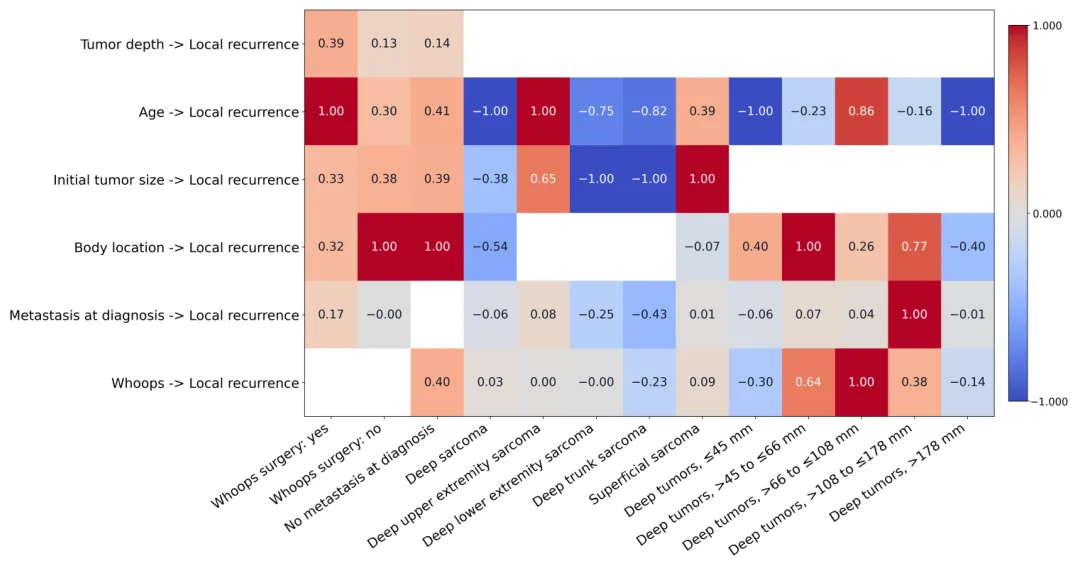
Normalized edge sensitivity scores for clinically relevant dependencies in the weighted interventional BN across exploratory clinical scenarios. Each score is the change in the estimated local recurrence ATT after marginalizing the parent variable from the corresponding child node conditional probability table, divided by the largest absolute change across the displayed scenario edge combinations. The remaining fitted network was unchanged. Scores range from −1 to +1, quantify sensitivity to modeled dependencies, and should not be interpreted as raw risk differences or causal effects of individual edges.

Figure 6 summarizes the most clinically relevant edge sensitivities across the scenario analyses. The figure is not intended to display every dependency to which the estimate was sensitive. Instead, it focuses on modeled dependencies related to local recurrence that are clinically interpretable.

The strongest dependency sensitivities varied across clinical scenarios. In patients with a previous whoops procedure, the dependencies with the largest sensitivities involved initial tumor size, body location, and tumor depth. In deep sarcomas, initial tumor size, whoops surgery status, and body location showed the largest sensitivities, while the age dependency had smaller sensitivity. The initial tumor size dependency also had smaller sensitivity in superficial sarcomas. The scenarios restricting deep sarcomas by tumor size or body location showed mixed patterns, with the age dependency showing larger sensitivity in several restricted scenarios despite its smaller sensitivity in the overall deep sarcoma scenario. This suggests that the modeled local recurrence contrast remained sensitive to dependencies involving tumor extent and anatomical context after stratifying by clinical scenario.

The edge sensitivity analysis should be interpreted as sensitivity of the fitted interventional contrast to specific modeled conditional dependencies, not as direct evidence that the parent variable causally changes the child variable. For example, dependencies involving body location, tumor size, tumor depth, whoops surgery status, metastasis at diagnosis, and local recurrence may reflect a combination of tumor extent, anatomical complexity, treatment planning, surgical decision making, pathological assessment, and residual structure in the registry data. Therefore, the analysis is best understood as an interpretability and robustness tool for the BN rather than as an independent causal effect estimate for each edge.

### 3.7. Robustness Analyses

Table 9 summarizes G3 conditioned estimates from models refitted in alternative populations. The conditioned refit medians were lower than the corresponding primary scenario estimates in 11 of the 13 alternative fitting cohorts, although the difference for deep trunk sarcoma was negligible (−0.01 pp), and higher in the superficial and 108–178 mm deep tumor cohorts. The largest downward shift occurred in deep tumors measuring >66 to ≤108 mm, while the largest upward shift occurred in superficial sarcoma. These changes indicate sensitivity to the fitting population rather than preservation of stable subgroup treatment effects.

The estimate for deep tumors measuring >66 to ≤108 mm changed direction after refitting, but every conditioned refit and primary analysis 95% perturbation interval included zero. Because no paired statistical comparison between the primary and refitted estimates was performed, the reported median differences quantify the direction and magnitude of the observed shifts but not whether they exceeded perturbation variability. More generally, these results highlight that extending the analysis to increasingly specific or sparsely supported subpopulations may introduce causal identification challenges, as changes in cohort composition, treatment assignment structure, and event sparsity can materially affect the stability of estimated effects. This underscores the importance of careful specification of the target population and estimand when interpreting conditional or stratified causal effects.

## 4. Discussion

A clinically specified causal BN distinguished treatment-associated patterns within the observed clinical pathway from contrasts under hypothetical treatment assignment. In this cohort, in which 99 of 151 patients received preoperative radiotherapy and treatment allocation varied by tumor size and depth, the primary weighted interventional BN had a bootstrap median ATT of +1.37 pp (95% percentile bootstrap CI, −3.19 to +5.81 pp), with the original sample estimate was +2.37 pp. Both summaries were small and close to the null, and the interval included estimates corresponding to modest reductions and modest increases in local recurrence. Accordingly, the near-null estimates should not be interpreted as evidence of equivalence or absence of a treatment effect. The bootstrap repeated patient sampling, propensity score estimation, weight construction, and BN parameter fitting while retaining the prespecified DAG. Conventional and weighted logistic regression had positive bootstrap medians of +3.3 and +5.2 pp, respectively. The movement toward the null after representing treatment assignment, downstream margin status, and local recurrence within the prespecified clinical pathway shows that the estimated contrast depended materially on how the treatment process was modeled. It should not, however, be interpreted as proof that one modeling framework recovers the true effect, because the BN and regression analyses make different structural and functional form assumptions.

These findings should be considered in relation to the established role of radiotherapy in local management of STS. Contemporary guidelines recommend radiotherapy for patients at increased risk of local recurrence, particularly for large, deep, high-grade tumors and when close or microscopically positive margins are anticipated or have occurred; treatment selection and sequencing should be determined in a multidisciplinary setting [2,13,14,16,17]. Randomized studies in extremity STS demonstrated improved local control when radiotherapy was added to limb-sparing surgery. Postoperative external-beam radiotherapy reduced local recurrence compared with surgery alone, including among patients with high-grade tumors, without demonstrating an overall survival advantage. Adjuvant brachytherapy after complete resection similarly improved local control, with benefit concentrated in high-grade tumors and no significant improvement in distant metastasis or survival [10,11]. A separate question concerns treatment timing. A randomized comparison of preoperative and postoperative radiotherapy demonstrated a higher risk of wound complications with preoperative treatment, but did not compare radiotherapy with its omission [12]. In a larger retrospective cohort of localized high-grade extremity STS, propensity score weighted analysis associated preoperative radiotherapy with a lower risk of local recurrence than no radiotherapy [15]. The present near-null estimate does not directly contradict the established evidence or support omission of radiotherapy because the studies differ in anatomical case mix, treatment eligibility, outcome analysis, and target estimand: our ATT applies specifically to surgically treated G3 patients managed without chemotherapy who actually received preoperative radiotherapy.

The contrast between the regression and interventional BN estimates also illustrates the clinical problem of confounding by indication. Patients selected for preoperative radiotherapy commonly have features that increase baseline local failure risk, so a positive observational recurrence contrast can persist even when radiotherapy is beneficial, neutral, or harmful. Explicitly modeling this selection pathway moved the estimate toward the null in the present data. This methodological finding supports careful representation of treatment selection when registry data are used to evaluate radiotherapy, but the near-null point estimate is not evidence of therapeutic equivalence.

The direct edge sensitivity analysis further showed that the estimated intervention contrast depended strongly on the assumed graph structure. When a clinically plausible direct radiotherapy to local recurrence edge was added, the unweighted and weighted interventional bootstrap medians shifted to −14.33 and −12.26 pp, respectively, and their intervals widened markedly, while the corresponding conditional estimates changed little. This pattern is consistent with the direct pathway becoming active under intervention while treatment simultaneously adds another parent to the local recurrence CPD. Because that addition approximately doubled the number of local recurrence conditional configurations in a cohort with only 31 recurrence events, the augmented model was substantially more weakly supported by the observed data. The resulting negative estimates therefore indicate structural sensitivity rather than reliable evidence of a direct protective effect of radiotherapy.

The exploratory scenarios likewise did not identify a clinically actionable subgroup. Every central 95% CPD perturbation interval for the weighted interventional estimates included zero, no consistent increase in benefit was observed with increasing tumor size, and estimates from smaller or restricted cohorts were unstable under refitting. Accordingly, these analyses do not provide robust evidence that any examined subgroup benefits from or is harmed by preoperative radiotherapy. Their appropriate role is to show sensitivity to case mix, treatment overlap, and model specification rather than to support subgroup-specific treatment recommendations.

The binary recurrence analysis did not directly account for the markedly different follow-up durations between treatment groups. In particular, shorter follow-up after surgery alone could result in fewer observed recurrences because these patients had less opportunity for recurrence detection. We therefore specified an additional time-to-event analysis in which the ATT treatment weights were multiplied by IPCW. At 1 year, the adjusted cumulative incidence difference was +0.83 percentage points (95% CI, −9.88 to +8.51), with similar estimated risks under preoperative radiotherapy (5.89%) and surgery alone (5.07%). The complementary time-to-event analysis yielded a similarly small point estimate. However its wide confidence interval and limited effective sample size preclude using it as independent validation of the BN estimate. This comparison should be interpreted as a complementary assessment because the BN estimates an overall binary recurrence contrast, while the time-to-event analysis estimates a risk contrast at a defined postoperative time.

The clinical implication is therefore not that preoperative radiotherapy can be omitted. The primary bootstrap confidence interval and the CPD perturbation intervals from the sensitivity analyses spanned estimates corresponding in direction and magnitude to clinically relevant benefit and harm, and recurrence ascertainment was especially limited by the median follow-up of 0.57 years after surgery alone compared with 2.76 years after preoperative radiotherapy; among patients who recurred, the median time to local recurrence was approximately 1.78 years. Shorter observation after surgery alone could undercount recurrences in that group and distort a binary comparison. Decisions should continue to integrate expected time varying risk, anticipated margins, anatomical site, resectability, and the competing short- and long-term toxicities of treatment within a sarcoma multidisciplinary team. The present framework may inform such evaluation by exposing the assumptions linking selection, treatment, surgery, and recurrence, but it is not a validated patient-level decision tool.

The edge sensitivity analysis has a narrower interpretive role. It showed that the fitted contrast was sensitive to clinically plausible dependencies involving tumor extent, anatomical context, and previous unplanned excision, but these scores are neither treatment effects nor evidence that individual variables causally alter recurrence. Together with the unstable restricted cohort estimates, this finding reinforces the need for external validation in larger cohorts with adequate treatment overlap and follow-up before considering individualized application.

### Strengths and Limitations

This study has several strengths. The causal question and target estimand were explicitly defined, and the clinically specified BN preserved the temporal ordering between baseline characteristics, treatment assignment, downstream surgical findings, and local recurrence. The network structure was defined a priori using clinical knowledge, while propensity score weighting was used to reduce measured treatment selection differences. In addition, the bootstrap propagated patient sampling, propensity score, weighting, and BN parameter estimation uncertainty through the analysis. Comparing regression, conditional BN, and interventional BN estimates, together with scenario and perturbation analyses, allowed the robustness of the findings to different modeling assumptions to be examined rather than relying on a single estimate.

Several limitations should also be considered. First, the retrospective observational design means that residual confounding from unmeasured or imperfectly measured factors cannot be excluded. Histologic subtype was reported descriptively but was not included in the primary adjustment because sparse histology combinations substantially reduced overlap and effective sample size; consequently, residual confounding by histology remains possible. Second, limited treatment overlap in some regions of the covariate distribution reduces the information available for estimating counterfactual outcomes and makes the results more dependent on modeling assumptions. Third, the modest overall sample size and limited number of local recurrence events constrain statistical precision, not only in the exploratory subgroups but also in the primary analysis. Radiotherapy dose and fractionation were unavailable for some treated patients, and completion of the planned course remained unverifiable for 3 of 99 patients; the binary treatment definition therefore did not capture possible variation in treatment intensity. Differential follow-up between treatment groups may also affect the ascertainment of local recurrence. The additional IPCW analysis addresses this problem only under the assumptions that censoring is conditionally independent of local recurrence given the measured variables, that each relevant patient profile has a positive probability of remaining under observation, and that the censoring model is correctly specified. Because the propensity score weights were held fixed during bootstrap resampling for the IPCW analysis, its reported confidence interval did not incorporate uncertainty from propensity score estimation. In contrast, the primary BN bootstrap reestimated the propensity score and ATT weights but retained the prespecified DAG. The alternative direct edge graph assessed one clinically important structural choice, but it did not quantify uncertainty over the broader set of plausible graph structures. IPCW cannot recover follow-up information where empirical support is absent and may become unstable when few patients remain under observation; the primary time-to-event estimand was therefore restricted to 1 year. Categorization of continuous variables necessarily entails loss of information and may introduce sensitivity to the selected cut-points, particularly in a small cohort with few recurrence events. However, sensitivity analyses using clinically interpretable age and tumor size categories, together with perturbations of those thresholds, produced similar treatment contrasts and did not alter the overall interpretation. The clinically specified BN structure also introduces modeling assumptions that may influence the estimated effects. In particular, the absence of a direct edge from preoperative radiotherapy to local recurrence in the primary graph assumes that the modeled effect operates through pathological margin status, although this coarse variable may not capture all biological mechanisms through which radiotherapy influences local control. Conversely, adding the direct edge approximately doubled the local recurrence CPD parameterization, produced sparse parent configurations, and materially changed the interventional estimates and their uncertainty. The two specifications therefore expose graph structure uncertainty that this modest dataset cannot resolve. Although no patient satisfying the treatment pathway criteria and having a recorded definitive resection margin had an R2 or other margin status, requiring an available margin assessment may still introduce selection if margin documentation was related to treatment or prognosis. Finally, restricting the study population to surgically treated G3 STS patients without chemotherapy limits the generalizability of the findings to broader sarcoma populations, including patients receiving perioperative systemic therapy. Taken together, the modest sample size and event count, limited treatment overlap, possible residual confounding, and differential follow-up restrict the precision and causal interpretation of the estimated ATT and preclude excluding modest treatment effects in either direction.

Future research should extend this work in several directions. First, larger independent multicentre cohorts are needed to improve precision, strengthen treatment overlap, and validate the estimated treatment effects across different clinical settings. Second, richer characterization of baseline clinical and tumor related factors may reduce residual confounding by better representing variables involved in both treatment selection and local recurrence risk. Third, the primary graph in which the effect is mediated by margin status and alternative prespecified graphs containing a direct edge from preoperative radiotherapy to local recurrence should be compared in substantially larger datasets with enough observations across local recurrence parent configurations to support the additional parameters. The causal framework could also be expanded beyond the present comparison of preoperative radiotherapy and surgery alone to represent the complete multimodal treatment pathway, including systemic therapy, postoperative treatment strategies, surgical findings, and subsequent disease management. Finally, with sufficiently detailed longitudinal data, dynamic Bayesian networks could extend the current static representation by modeling the evolution of patient state, treatment decisions, and outcomes over time.

## 5. Conclusions

In this selected cohort of surgically treated G3 STS patients managed without chemotherapy, the primary weighted interventional BN had a bootstrap median ATT of +1.37 pp, with a 95% percentile bootstrap CI from −3.19 to +5.81 pp, while the original sample estimate was +2.37 pp. Because the bootstrap distribution spanned contrasts corresponding to modest benefit and modest harm, the near-null estimates should not be interpreted as evidence of equivalence or absence of a treatment effect. Conventional regression produced positive contrasts, while explicit representation of treatment selection and the clinical pathway moved the estimate toward the null. Adding a direct radiotherapy to local recurrence edge shifted the weighted interventional bootstrap median to −12.26 pp but substantially increased uncertainty because the augmented local recurrence CPD was sparsely supported. The bootstrap quantified sampling uncertainty for the primary and direct edge analyses, while CPD perturbation was used for the remaining sensitivity and robustness analyses, which identified no consistent or robust subgroup treatment effect pattern.

The one-year ATT × IPCW analysis yielded a similarly small point estimate but had substantial uncertainty and limited effective sample size. It should therefore be interpreted as a complementary analysis rather than as validation of the primary BN result.

These findings do not support changing established indications for preoperative radiotherapy or omitting it in patients at increased risk of local recurrence. Rather, they show how confounding by indication, unequal follow-up, and clinical pathway assumptions can materially shape observational estimates. Given the modest sample size, limited treatment overlap, differential follow-up, and potential residual confounding, these causal estimates should be interpreted cautiously and require confirmation in larger, more completely followed multicentre cohorts before this framework can inform individual treatment decisions.

## Figures and Tables

**Table 1:** Radiotherapy classification rules used for the primary causal comparison.

Clinical Situation	Classification	Detail
Planned biopsy followed by preoperative radiotherapy and definitive resection	Preoperative radiotherapy	Radiotherapy occurs before definitive oncologic surgery
Whoops surgery followed by radiotherapy and definitive repeat resection	Preoperative radiotherapy	Whoops is treated as a covariate, and radiotherapy is preoperative relative to definitive oncologic surgery
Surgery alone	Surgery alone	Definitive surgery without preoperative radiotherapy
Definitive resection followed by postoperative radiotherapy	Excluded	Postoperative radiotherapy represents a distinct treatment pathway

**Table 2:** Characteristics and data completeness of the primary G3 cohort (n=151). Binary and categorical variables are reported as number and percentage of the full cohort unless otherwise specified. Continuous variables were discretized using quantile binning. Missingness was retained as an explicit model category where applicable.

Variable	Observed	Missing	Observed Distribution/Discretization
Baseline characteristics			
Whoops surgery	151	0	Yes: 39 (25.8%); No: 112 (74.2%)
Metastasis at diagnosis	151	0	Yes: 6 (4.0%); No: 145 (96.0%)
Histologic subtype	151	0	Undifferentiated sarcoma: 47 (31.1%); Liposarcoma: 35 (23.2%); Fibroblastic tumor: 23 (15.2%); Leiomyosarcoma: 19 (12.6%); Vascular sarcoma: 11 (7.3%); Other histologies: 16 (10.5%)
Age	151	0	≤59 years: 39 (25.8%); >59 to ≤70 years: 39 (25.8%); >70 to ≤77 years: 36 (23.8%); >77 years: 37 (24.5%)
Body location	151	0	Upper extremity: 32 (21.2%); Lower extremity: 32 (21.2%); Trunk: 87 (57.6%)
Tumor depth	151	0	Deep: 111 (73.5%); Superficial: 40 (26.5%)
Initial tumor size	151	0	≤45 mm: 32 (21.2%); >45 to ≤66 mm: 29 (19.2%); >66 to ≤108 mm: 30 (19.9%); >108 to ≤178 mm: 30 (19.9%); >178 mm: 30 (19.9%)
Treatment assignment			
Preoperative radiotherapy	151	0	Yes: 99 (65.6%); No: 52 (34.4%)
Downstream surgical finding			
Pathological margin status	151	0	R0: 121 (80.1%); R1: 30 (19.9%)
Outcome			
Local recurrence	151	0	Yes: 31 (20.5%); No: 120 (79.5%)

**Table 3:** Temporal classification and analytical use of study variables in the propensity score (PS) and BN models.

Clinical Timing	Variables	Used in PS	Used in BN
Baseline at treatment decision time zero	Age, metastasis at diagnosis, body location, depth, initial tumor size, whoops surgery	Yes	Yes
Baseline descriptive variable	Histologic subtype	No	No
Treatment decision	Preoperative radiotherapy	No	Yes
Surgical finding after treatment	Pathological margin status	No	Yes
Postoperative outcome (time origin: definitive resection)	Local recurrence	No	Yes

**Table 4:** Summary propensity score diagnostics for the G3 ATT weighted causal comparison. SMD = standardized mean difference.

Diagnostic	Before Weighting	After Weighting
Maximum absolute SMD	0.866	0.139
Mean absolute SMD	0.329	0.090
Adjustment covariates with |SMD|>0.15	6/8	0/8
G3 weighting sample size/ESS	n=151	80.6
Propensity score overlap	Common support 0.362–0.895	10 rows outside common support
Weight distribution	–	median 1.000; p99 7.641; max 7.989

**Table 5:** Sensitivity of the weighted interventional BN estimate to weight truncation and common support restriction.

Analysis	N	Treated / SA	ESS	Maximum Absolute SMD	Median BN ATT, pp	Bootstrap 95% CI, pp
99th percentile truncation (Primary)	151	99 / 52	80.6	0.139	+1.4	−3.2 to +5.8
Untruncated	151	99 / 52	61.9	0.143	+1.8	−3.0 to +5.6
Common support restriction	141	89 / 52	72.4	0.183	+2.1	−3.9 to +6.0

**Table 6:** Graph structure sensitivity analysis adding a direct edge from preoperative radiotherapy to local recurrence. Estimates are risk differences for preoperative radiotherapy minus surgery alone in pp. Negative values indicate lower estimated local recurrence risk under preoperative radiotherapy.

BN Analysis	Bootstrap Median, pp	95% Bootstrap CI, pp
Bayesian conditioning	−5.16	−9.55 to −0.37
Bayesian intervention	−14.33	−28.73 to −2.68
Weighted Bayesian conditioning	−3.19	−9.30 to +2.13
Weighted Bayesian intervention	−12.26	−25.57 to −0.83

**Table 7:** Bayesian network CPD perturbation sensitivity results for the primary cohort and exploratory clinical scenarios. Values are perturbation distribution median risk differences in pp with central 95% CPD perturbation intervals and therefore differ from the original sample estimates and percentile bootstrap confidence intervals in Figure 4. These are parameter sensitivity intervals, not confidence or posterior credible intervals. Positive values indicate a higher estimated local recurrence risk under preoperative radiotherapy, while negative values indicate a lower estimated risk.

Scenario	*n*	RT	Surg.	BN Cond.	BN Interv.	Weighted BN Cond.	Weighted BN Interv.
Primary cohort	151	99	52	−1.2(−9.2,+10.5)	−0.4(−9.3,+10.2)	+1.6(−6.4,+7.7)	+1.6(−5.3,+9.3)
Exploratory scenarios							
Whoops surgery: yes	39	26	13	−0.1(−5.6,+5.1)	+0.0(−2.8,+2.5)	+0.6(−3.5,+5.4)	+0.5(−1.4,+3.7)
Whoops surgery: no	112	73	39	−3.7(−10.0,+1.8)	+0.0(−4.8,+3.7)	−2.3(−7.3,+4.0)	+1.0(−2.2,+5.0)
No metastasis at diagnosis	144	95	49	−2.8(−10.7,+2.2)	−0.0(−4.5,+3.9)	−1.9(−6.8,+3.9)	+1.1(−2.2,+4.5)
Deep sarcoma	111	77	34	−3.6(−9.0,+1.6)	+0.0(−4.6,+3.5)	−1.9(−7.4,+3.9)	+1.0(−2.2,+4.4)
Deep upper extremity sarcoma	26	24	2	−5.9(−15.7,+1.6)	−0.0(−4.5,+3.7)	−4.3(−13.5,+4.1)	+0.9(−2.2,+5.4)
Deep lower extremity sarcoma	22	18	4	−2.4(−13.3,+5.1)	−0.0(−1.8,+1.9)	−3.9(−13.2,+6.2)	+0.8(−1.9,+4.8)
Deep trunk sarcoma	63	35	28	−3.1(−11.3,+3.7)	+0.0(−5.5,+4.2)	−0.0(−7.0,+7.3)	+0.9(−2.6,+5.0)
Superficial sarcoma	40	22	18	−0.4(−6.6,+4.9)	+0.0(−2.9,+3.4)	−0.9(−7.2,+3.9)	+0.6(−1.2,+4.1)
Deep tumors, ≤45 mm	12	8	4	+0.0(−8.2,+7.8)	−0.0(−4.2,+4.2)	+0.8(−7.3,+8.3)	+0.9(−3.0,+6.0)
Deep tumors, >45 to ≤66 mm	18	13	5	−4.7(−15.7,+4.1)	+0.0(−3.8,+3.4)	−3.8(−17.5,+7.7)	+0.4(−2.1,+4.6)
Deep tumors, >66 to ≤108 mm	26	20	6	−7.3(−17.7,+4.1)	−0.0(−6.9,+7.3)	−7.2(−17.6,+4.1)	+1.6(−3.3,+8.2)
Deep tumors, >108 to ≤178 mm	28	22	6	−4.1(−13.3,+5.8)	−0.1(−9.0,+8.0)	−3.0(−13.4,+5.7)	+1.1(−2.1,+8.1)
Deep tumors, >178 mm	26	13	13	−0.5(−11.4,+9.8)	+0.0(−1.6,+2.2)	+3.0(−8.7,+15.8)	+0.2(−1.5,+3.6)

**Table 8:** Diagnostics for IPCW and combined ATT × IPCW analysis weights among patients remaining at risk at 1 year. Weight summaries are pooled across treatment groups.

Diagnostic at 1 Year	IPCW	Combined ATT × IPCW
Median	1.229	1.229
95th percentile	2.261	2.607
99th percentile	2.619	16.084
Maximum	2.696	19.034
Effective sample size	84.539	28.129
Number remaining at risk: preoperative RT	69	
Number remaining at risk: surgery alone	21	

**Table 9:** Robustness analysis of the conditioned on G3 weighted BN intervention estimate across alternative model fitting cohorts. Estimates are reported as median ATT risk differences in percentage points, with the 2.5th and 97.5th percentiles of the perturbed CPD distribution in brackets. These empirical 95% perturbation intervals summarize parameter sensitivity and are not confidence or posterior credible intervals. The difference column represents the median from the conditioned refit minus the corresponding estimate from the primary analysis. Positive values indicate a higher estimated local recurrence risk under preoperative radiotherapy.

Fitting Cohort	Conditioned on G3 ATT	Primary Analysis ATT	Median Difference
Whoops surgery: yes	+0.12[−1.00,+1.37]	+0.50[−1.40,+3.70]	−0.38
Whoops surgery: no	−0.10[−3.22,+3.49]	+1.00[−2.20,+5.00]	−1.10
No metastasis at diagnosis	+0.12[−1.71,+2.72]	+1.10[−2.20,+4.50]	−0.98
Deep sarcoma	+0.74[−1.84,+3.54]	+1.00[−2.20,+4.40]	−0.26
Deep upper extremity sarcoma	+0.20[−2.36,+3.48]	+0.90[−2.20,+5.40]	−0.70
Deep lower extremity sarcoma	0.00[−1.42,+1.92]	+0.80[−1.90,+4.80]	−0.80
Deep trunk sarcoma	+0.89[−2.54,+6.16]	+0.90[−2.60,+5.00]	−0.01
Superficial sarcoma	+0.86[−0.39,+3.16]	+0.60[−1.20,+4.10]	+0.26
Deep tumors, ≤45 mm	0.00[−0.40,+0.63]	+0.90[−3.00,+6.00]	−0.90
Deep tumors, >45 to ≤66 mm	+0.09[−0.62,+1.54]	+0.40[−2.10,+4.60]	−0.31
Deep tumors, >66 to ≤108 mm	−0.50[−2.46,+0.95]	+1.60[−3.30,+8.20]	−2.10
Deep tumors, >108 to ≤178 mm	+1.24[−1.15,+4.80]	+1.10[−2.10,+8.10]	+0.14
Deep tumors, >178 mm	+0.01[−0.98,+1.29]	+0.20[−1.50,+3.60]	−0.19

## Data Availability

Restrictions apply to the availability of these data because they contain confidential clinical registry information. Data may be available upon reasonable request and with permission from the Swiss Sarcoma Network and the relevant data governance bodies.

## Abbreviations

The following abbreviations are used in this manuscript:

ATT	Average treatment effect among treated patients
BASEC	Business Administration System for Ethics Committees
BN	Bayesian network
CI	Confidence interval
CPD	Conditional probability distribution
EM	Expectation–maximization
ESS	Effective sample size
FNCLCC	French Federation of Cancer Centers Sarcoma Group
G3	Histologic grade 3
IPCW	Inverse probability of censoring weighting
LR	Local recurrence
pp	Percentage points
PS	Propensity score
R0	Microscopically margin negative resection
R1	Microscopically margin positive resection
SMD	Standardized mean difference
STS	Soft tissue sarcoma
